# Apoptosis-related factors are relevant to progression of pancreatic neuroendocrine tumors

**DOI:** 10.1186/s12957-023-03267-4

**Published:** 2023-12-12

**Authors:** Shota Amano, Teijiro Hirashita, Yoko Kawano, Haruto Nishida, Hiroki Orimoto, Masahiro Kawamura, Takahide Kawasaki, Takashi Masuda, Yuichi Endo, Masayuki Ohta, Tsutomu Daa, Masafumi Inomata

**Affiliations:** 1 Department of Gastroenterological and Pediatric Surgery, Oita University Faculty of Medicine, 1-1 Idaigaoka, Hasama-Machi, Yufu, Oita 879-5593 Japan; 2 Department of Diagnostic Pathology, Oita University Faculty of Medicine, Oita, Japan; 3 Department of Comprehensive Surgery for Community Medicine, Oita University Faculty of Medicine, Oita, Japan; 4 Global Oita Medical Advanced Research Center for Health, Oita University, Oita, Japan

**Keywords:** Caspase3, Pancreatic neuroendocrine tumors, Progression, Survivin, XIAP

## Abstract

**Background:**

Multidisciplinary therapy centered on antitumor drugs is indicated in patients with unresectable pancreatic neuroendocrine tumors (PanNET). However, the criteria for selection of optimal therapeutic agents is controversial. The aim of this study was to assess the malignancy of PanNET for optimal therapeutic drug selection.

**Methods:**

Forty-seven patients with PanNET who underwent surgery were reviewed retrospectively, and immunohistochemical characteristics, including expression of GLUT1, SSTR2a, SSTR5, Survivin, X-chromosome-linked inhibitor of apoptosis protein (XIAP), and Caspase3 in the resected specimens, were investigated. Relapse-free survival (RFS) and overall survival (OS) were evaluated with regard to the characteristics using the Kaplan–Meier method and compared with the log-rank test.

**Results:**

GLUT1 expression showed significant correlation with sex (*p* = 0.036) and mitotic rate (*p* = 0.048). Survivin and XIAP expression showed significant correlation with T-stage (*p* = 0.014 and 0.009), p-Stage (*p* = 0.028 and 0.045), and mitotic rate (*p* = 0.023 and 0.007). XIAP expression also significantly influenced OS (*p* = 0.044).

**Conclusions:**

Survivin and XIAP correlated with grade of malignancy, and expression of XIAP in particular was associated with a poor prognosis. Expression of these proteins may be a useful indicator to select optimal therapeutic agents in PanNET.

**Supplementary Information:**

The online version contains supplementary material available at 10.1186/s12957-023-03267-4.

## Background

Pancreatic neuroendocrine tumors (PanNET) are thought to arise from the endocrine cells of the pancreas [1]. PanNET can cause various symptoms because they may produce hormones such as insulin, gastrin, glucagon, vasoactive intestinal peptide, and somatostatin [2]. The number of patients with PanNET has been rising worldwide in recent years [3], and in Japan, the incidence rate of PanNET in 2020 was estimated to be 0.70 per 100,000 people [4]. PanNET are generally slow-growing [5]. However, among the patients with PanNET, 23.2% exhibited distant metastases at the time of diagnosis [4]. As the grade increased, the percentage of patients with distant metastasis also increased at initial diagnosis. Distant metastasis occurred in 5.8% of patients with a neuroendocrine tumor (NET) of grade G1 and in 59.7% with a NET of grade G3 or neuroendocrine carcinoma [4]. Therefore, it is important to accurately evaluate the tumor as treatment should be based on its functionality, degree of progression, and presence or absence of metastases.

Surgical resection is the only curative treatment and is recommended if the preoperative diagnosis indicates this possibility. In contrast, multidisciplinary therapy centered on antitumor drugs is indicated in unresectable cases. To date, drug therapies for PanNET include somatostatin analogues, molecular-targeted agents, and cytotoxic agents, and peptide receptor radionuclide therapy (PRRT) as also listed as a treatment option [6]. Five subtypes of SSTRs have been found [7] and SSTR2a and SSTR5 are considered therapeutic targets because of the frequent expression in PanNET. Everolimus, a mammalian target of rapamycin (mTOR) inhibitor, and Sunitinib, a tyrosine kinase inhibitor, are molecular targeted therapies used in PanNET. Everolimus suppress anti-apoptotic molecule Bcl-2 function and apoptosis proceeds [8]. Sunitinib decreases its downstream effector protein kinase B (Akt)/mTOR/ribosomal protein S6 kinase 1 (S6K1) by inhibiting vascular endothelial growth factor receptor-2 (VEGFR-2) [9]. As a result, the anti-apoptotic molecule Bcl-2 activity is inhibited, which may exert an apoptotic effect. Yao et al. [1] and Ikeda et al. [10] suggested that treatment strategy is decided according to tumor burden and aggressiveness. The clinical guidelines published in ESMO 2020 recommended that the treatment strategy might be decided using the grade classified by the mitotic rate and Ki-67 index and the somatostatin receptor (SSTR) expression [6]. However, criteria for selection of optimal therapeutic agents is still controversial.

The mitotic rate and Ki-67 index are indicators of cell proliferation ability. However, factors that inhibit proliferation can also act in some way during the process of tumor cell growth. Apoptosis is one of the functions that inhibits tumor cell proliferation, and avoidance of apoptosis is also considered to be involved in malignancy. But how apoptosis acts in PanNET remains unclear. Therefore, we hypothesized that the function of apoptosis may affect cell proliferation and may also be related to malignancy in PanNET. Cell apoptosis is regulated by cysteine-aspartic acid protease family, caspases [11]. Among them, Caspase3, also known as execution caspase, plays an important role in apoptosis. Survivin and X-chromosome-linked inhibitor of apoptosis protein (XIAP) are inhibitors of apoptosis proteins (IAP) family members [12]. Functionally, Survivin inhibits apoptosis by binding to XIAP and inhibiting the activity of Caspase3. Furthermore, in malignant cells, cell proliferative capacity correlates with glucose metabolism, and GLUT1 is overexpressed in many carcinomas [13, 14]. Therefore, GLUT1 was also included in this study to assess the malignancy of PanNET.

The aim of this study was thus to investigate the relationship between the expression of GLUT1, SSTRs (SSTR2a and SSTR5), and apoptotic regulators (Survivin, XIAP, and Caspase3) and clinicopathological factors using immunohistochemical staining, and to assess the malignancy of PanNET for optimal therapeutic drug selection.

## Methods

From 2001 to 2023, 47 patients who underwent surgery for PanNET at the Department of Gastroenterological and Pediatric Surgery, Oita University were enrolled in this study. Formalin-fixed paraffin-embedded tissue blocks showing representative histology were selected for each case and cut at a thickness of 4 µm. Histological features were assessed with hematoxylin–eosin staining and immunohistochemistry (chromogranin A [CgA], synaptophysin, CD56, and Ki-67) from formalin-fixed, paraffin-embedded tissues. According to the 2019 WHO classification, tumor grade is classified to grade 1 to 3 using mitotic rate of the tumor cells and/or the Ki-67 proliferation index [5]. According to the 8th edition of the Union for International Cancer Control (UICC) tumor, nodes, and metastases (TNM) Classification, p-Stage classification was performed. Clinical characteristics were examined retrospectively. This research protocol received the approval of the institutional ethics committee and review board of Oita University (approval number: 2540) and conformed to the guidelines of the Declaration of Helsinki.

For immunohistochemical staining, we used the biotin-streptavidin method and a Histofine kit (Nichirei, Tokyo, Japan). The antibodies we used are listed in Table 1. Briefly, the sections were deparaffinized in xylene and rehydrated in graded alcohol. Endogenous peroxidase activity was abolished by incubation with 3% hydrogen peroxide for 20 min at room temperature. Antigen retrieval was performed by placing the slides in a citrate buffer of pH 6.0 or 9.0 and heating them in an autoclave at 121ºC for 15 min. These slides were further incubated with primary antibodies overnight in a moist chamber at 4ºC. The immunoreaction was visualized using 3,3’-diaminobenzidine solution for 5 min. Finally, the slides were counterstained with hematoxylin.

**Table 1:** Primary antibodies and their conditioning in this study

Antibody	Clone	Dilution	Condition	Source
GLUT1	Rabbit monoclonal (EPR3915)	1/500	pH 6.0, overnight	Abcam, Cambridge, UK
Survivin	Rabbit polyclonal (BC008718)	1/200	pH 9.0, overnight	Proteintech, Rosemont, IL, USA
XIAP	Rabbit polyclonal (BC032729)	1/100	pH 9.0, overnight	Proteintech, Rosemont, IL, USA
Caspase3	Rabbit monoclonal (E87)	1/100	pH 9.0, overnight	Abcam, Cambridge, UK
SSTR2a	Rabbit monoclonal (UMB1)	1/200	pH 6.0, overnight	Abcam, Cambridge, UK
SSTR5	Rabbit monoclonal (UMB4)	1/200	pH 9.0, overnight	Abcam, Cambridge, UK
Ki-67	Mouse monoclonal (MM1)	Diluted	pH 6.0, overnight	Leica Biosystems, Newcastle, UK

The slides were reviewed by two independent pathologists (SA and HN in authors) who had no knowledge of patient outcomes. GLUT1 was considered positive when the cell membrane was stained [15]. The expressions of SSTR2a, and SSTR5 were considered positive when they were present in the membrane, the cytoplasm, or both [16]. Survivin and XIAP were positive when they were expressed in the cytoplasm and/or nucleus of the tumor cells [17]. Caspase3 expressed in the cytoplasm of the tumor cells was determined to be positive [11]. With reference to the evaluation method performed by Takada et al. [18], we determined that more than 30% reactivity in tumor cells indicated a positive result.

We included the following 16 clinicopathological factors in the analyses: age, sex, tumor location (pancreatic head, body and/or tail), operation (pancreatoduodenectomy: PD/distal pancreatectomy: DP/others), tumor size, vascular invasion, extrapancreatic invasion, functionality, UICC stage (T-stage/N-stage/M-stage/ p-Stage), mitotic rate, Ki-67 index, grade (WHO classification 2019), and recurrence. All variables are expressed as mean ± standard deviations for continuous data. To evaluate the relation between clinicopathological and immunohistochemical variables, comparisons between groups were assessed by using the chi-squared test or Fischer’s exact test.

Relapse-free survival (RFS) was considered to be the period from resection to the first radiological evidence of tumor recurrence. Overall survival (OS) was defined as the period between the day of surgery until the date of death due to any cause or the day of last follow-up. RFS and OS were estimated with the Kaplan–Meier method, and the log-rank test was used to assess differences between groups.

Differences were regarded as significant when the *p* value was < 0.05. All statistical analyses were performed using SPSS statistics for Mac (version 28.0.1.0; SPSS Japan, Tokyo, Japan).

## Results

### Patient characteristics

The clinicopathological findings of the 47 cases retrieved from the patients’ medical records are summarized in Table 2. Mean patient age was 61.7 ± 13.9 years, 35 (74.5%) patients were women, and 12 (25.5%) were men. Tumors were located at the head of the pancreas in 13 patients (27.7%) and body and/or tail in 34 (72.3%). The mean diameter of the tumors was 21.4 ± 20.1 mm. The neuroendocrine neoplasms were functional 13 patients (27.7%) and non-functional in 34 (72.3%). Stage classification was p-Stage I in 31 cases (65.9%), p-Stage II in 10 (21.3%), p-Stage III in 4 (8.5%), and p-Stage IV in 2 (4.3%). Of the 47 patients, 5 (10.1%) had lymph node metastasis and 2 (4.3%) had distant metastasis. The Ki-67 index was < 3% in 39 patients (83.0%) and ≥ 3% in 8 patients (17%).

**Table 2:** Clinicopathological findings of the 47 cases of PanNET

Characteristics	*n* = 47
Age (years)	61.7 ± 13.9
Sex (Female/Male)	35 (74.5%)/12 (25.5%)
Tumor location (Ph/Pbt)	13 (27.7%)/34 (72.3%)
Operation (PD/DP/Others)	13 (27.7%)/30 (63.8%)/4 (8.5%)
Tumor size (mm)	21.4 ± 20.1
Vascular invasion (negative/positive)	41 (87.2%)/6 (12.8%)
Extrapancreatic invasion (negative/positive)	42 (89.4%)/5 (10.6%)
Functionality (non-functional/functional)	34 (72.3%)/13 (27.7%)
UICC Stage	
T-stage (T1/T2/T3/T4)	33 (70.2%)/10 (21.3%)/4 (8.5%)/0 (0%)
N-stage (N0/N1)	42 (89.4%)/5 (10.6%)
M-stage (M0/M1)	45 (95.7%)/2 (4.3%)
p-Stage (I/II/III/IV)	31 (65.9%)/10 (21.3%)/4 (8.5%)/2 (4.3%)
Mitoses / 2mm^2^ (< 2/ ≥ 2)	39 (83.0%)/8 (17.0%)
Ki-67 proliferation index (< 3%/ ≥ 3%)	39 (83.0%)/8 (17.0%)
Grade (G1/G2/G3)	37 (78.7%)/9 (19.1%)/1 (2.1%)
Recurrence (negative/positive)	41 (87.2%)/6 (12.8%)

*PanNET* pancreatic neuroendocrine tumors, *Ph* pancreatic head, *Pbt* pancreatic body and tail, *PD* pancreatoduodenectomy, *DP* distal pancreatectomy

The mitotic rate was < 2 in 39 patients (83.0%) and ≥ 2 in 8 patients (17%). Regarding tumor grade, 37 patients (78.7%) were diagnosed pathologically as having G1, 9 (19.1%) as G2, and 1(2.1%) as G3 tumors. CgA was positive for expression in all cases. Six patients (12.8%) suffered recurrence following surgery, with the location being the liver in 5 of the patients (83.3%) (Table 3). Tumor grade was G2 in 5 patients (83.3%), and 1 patient (16.7%) had a functional neuroendocrine tumor (insulinoma). The average time interval from resection to the diagnosis of tumor recurrence was 42.5 ± 20.5 months (range 9–72 months).

**Table 3:** Characteristics of the recurrence cases

Age (years)/Sex	Tumor location	Tumor size (mm)	Vascular invasion	Extrapancreatic invasion	Stage	Mitoses / 2mm^2^	Ki-67 proliferation index	Grade	Functionality	Site of recurrence	Time of recurrence (months)
73/m	Ph	27	Positive	Negative	II	2	1%	G2	Non-functional	Retroperitoneal	72
56/f	Pbt	18	Positive	Positive	III	10	5%	G2	Non-functional	Liver	48
76/m	Ph	32	Positive	Negative	III	1	2%	G1	Non-functional	Liver	60
58/f	Pbt	75	Negative	Negative	IV	2	5%	G2	Functional (insulinoma)	Liver	30
35/f	Pbt	60	Positive	Negative	IV	5	8%	G2	Non-functional	Liver	9
64/m	Pbt	27	Negative	Negative	II	3	4%	G2	Non-functional	Liver	36

*Ph* pancreatic head, *Pbt* pancreatic body and tail

### Relationships between immunohistochemical factors and clinicopathological characteristics

**Fig. 1:**
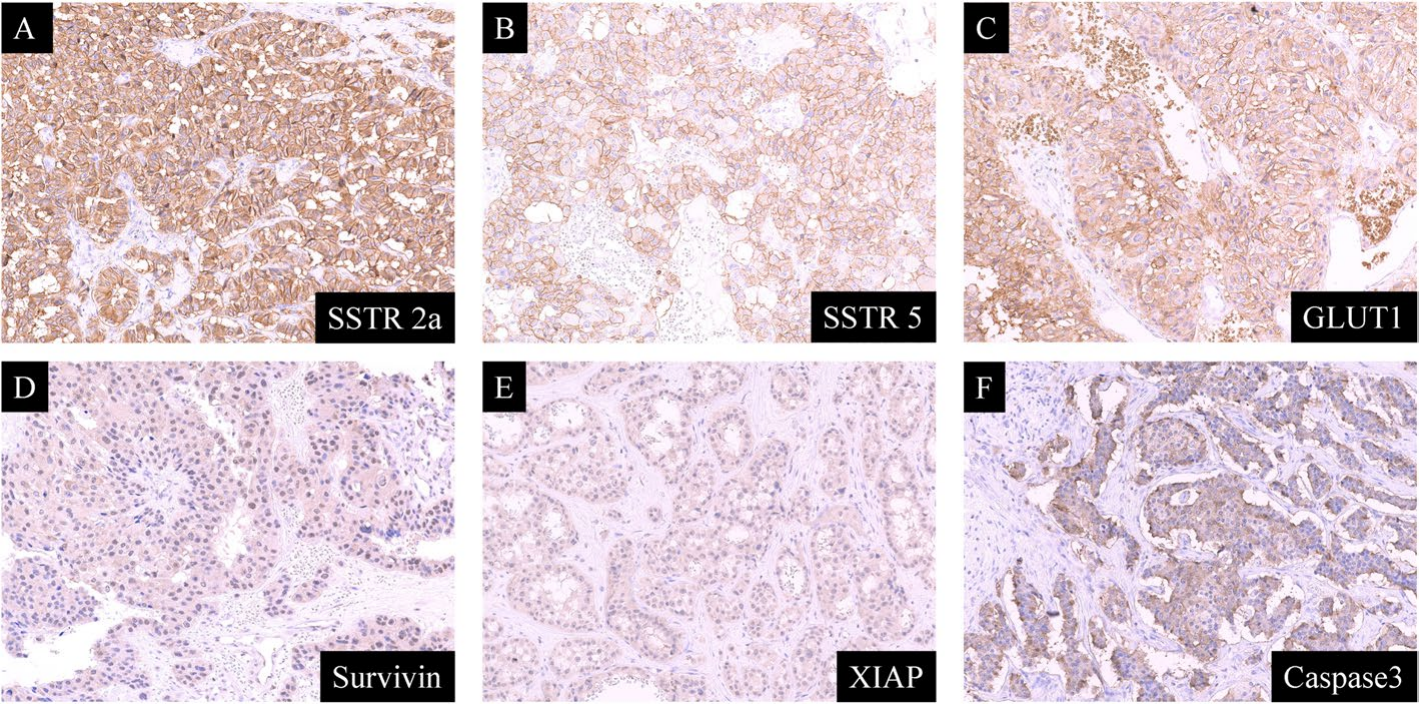
Representative photomicrographs of immunohistochemistry in pancreatic neuroendocrine tumors (PanNET) (original magnification, 200 ×). **A** Expression of SSTR2a, **B** Expression of SSTR5, **C** Expression of GLUT1, **D** Expression of Survivin, **E** Expression of XIAP, **F** Expression of Caspase3

Among the 47 cases, the numbers of patients positive for GLUT1, Survivin, XIAP, Caspase3, SSTR2a, and SSTR5 were 24 (51.1%), 23 (48.9%), 20 (42.6%), 38 (80.9%), 32 (68.1%), and 18 (38.3%), respectively. Representative photomicrographs of immunohistochemistry are shown in Fig. 1.

The relationships between the immunohistochemical factors and clinicopathological characteristics are shown in Table 4. The expression of GLUT1 was significantly associated with sex (female: 60.0% vs male: 25.0%, *p* = 0.036) and mitotic rate (< 2: 43.6% vs ≥ 2: 87.5%, *p* = 0.048). The expression of Survivin was significantly associated with T-stage (T1: 36.4% vs T2: 70.0% vs T3: 100%, *p* = 0.014), p-Stage (I: 38,7% vs II: 80.0% vs III: 25.0% vs IV: 100%, *p* = 0.028), and mitotic rate (< 2: 41.0% vs ≥ 2: 87.5%, *p* = 0.023). The expression of XIAP was significantly associated with T-stage (T1: 30.3% vs T2: 60.0% vs T3: 100%, *p* = 0.009), p-Stage (I: 32.3% vs II: 70.0% vs III: 25.0% vs IV: 100%, *p* = 0.045), and mitotic rate (< 2: 33.3% vs ≥ 2: 87.5%, *p* = 0.007). The expression of Caspase3 was significantly associated with T-stage (T1: 90.9% vs T2: 50.0% vs T3: 75.0%, *p* = 0.012), p-Stage (I: 90.3% vs II: 50.0% vs III: 75.0% vs IV: 100%, *p* = 0.031), mitotic rate (< 2: 87.2% vs ≥ 2: 50.0%, *p* = 0.033), and Grade (G1: 86.5% vs G2: 66.7% vs G3: 0%, *p* = 0.040). There was no relationship between the expression of SSTR2a or SSTR5 and any of the clinicopathological characteristics.

**Table 4:** Correlation between the expression of investigated proteins and clinicopathological data

Clinicopathological data, n (%)		GLUT1		SSTR2a		SSTR5		Survivin		XIAP		Caspase3	
	n	Positive	*p* value	Positive	*p* value	Positive	*p* value	Positive	*p* value	Positive	*p* value	Positive	*p* value
Age, years													
< 62	18	9 (50.0)	0.908	15 (83.3)	0.077	8 (44.4)	0.495	12 (66.7)	0.055	10 (55.6)	0.155	12 (66.7)	0.068
≥ 62	29	15 (51.7)		17 (58.6)		10 (34.5)		11 (37.9)		10 (34.5)		26 (89.7)	
Sex													
Female	35	21 (60.0)	0.036	23 (65.7)	0.725	12 (34.3)	0.493	19 (54.3)	0.21	14 (40.0)	0.545	29 (82.9)	0.674
Male	12	3 (25.0)		9 (75.0)		6 (50.0)		4 (33.3)		6 (50.0)		9 (75.0)	
Functionality													
Non-functional	34	17 (50.0)	0.813	21 (61.8)	0.175	13 (38.2)	1	16 (47.1)	0.677	15 (44.1)	0.726	27 (79.4)	1
Functional	13	7 (53.8)		11 (84.6)		5 (38.5)		7 (53.8)		5 (38.5)		11 (84.6)	
Tumor size, mm													
< 21	33	18 (54.5)	0.464	22 (66.7)	1	12 (36.4)	0.675	12 (36.4)	0.008	10 (30.3)	0.009	30 (90.9)	0.013
≥ 21	14	6 (42.9)		10 (71.4)		6 (42.9)		11 (78.6)		10 (71.4)		8 (57.1)	
Vascular invasion													
Negative	41	20 (48.8)	0.666	28 (68.3)	1	16 (39.0)	1	20 (48.8)	1	17 (41.5)	1	33 (80.5)	1
Positive	6	4 (66.7)		4 (66.7)		2 (33.3)		3 (50.0)		3 (50.0)		5 (83.3)	
Extrapancreatic invasion													
Negative	42	19 (45.2)	0.05	28 (66.7)	1	17 (40.5)	0.636	21 (50.0)	1	18 (42.9)	1	35 (83.3)	0.24
Positive	5	5 (100)		4 (80.0)		1 (20.0)		2 (40.0)		2 (40.0)		3 (60.0)	
T-stage													
T1	33	18 (54.5)	0.245	22 (66.7)	0.408	12 (36.4)	0.895	12 (36.4)	0.014	10 (30.3)	0.009	30 (90.9)	0.012
T2	10	3 (30.0)		6 (60.0)		4 (40.0)		7 (70.0)		6 (60.0)		5 (50.0)	
T3	4	3 (75.0)		4 (100)		2 (50.0)		4 (100)		4 (100)		3 (75.0)	
N-stage													
N0	42	21 (50.0)	1	28 (66.7)	1	16 (38.1)	1	21 (50.0)	1	18 (42.9)	1	34 (81.0)	1
N1	5	3 (60.0)		4 (80.0)		2 (40.0)		2 (40.0)		2 (40.0)		4 (80.0)	
M-stage													
M0	45	22 (48.9)	0.489	30 (66.7)	1	17 (37.8)	1	21 (46.7)	0.234	18 (40.0)	0.176	36 (80.0)	1
M1	2	2 (100)		2 (100)		1 (50.0)		2 (100)		2 (100)		2 (100)	
p-Stage													
I	31	17 (54.8)	0.279	21 (67.7)	0.889	12 (38.7)	1	12 (38.7)	0.028	10 (32.3)	0.045	28 (90.3)	0.031
II	10	3 (30.0)		6 (60.0)		4 (40.0)		8 (80.0)		7 (70.0)		5 (50.0)	
III	4	2 (50.0)		3 (75.0)		1 (25.0)		1 (25.0)		1 (25.0)		3 (75.0)	
IV	2	2 (100)		2 (100)		1 (50.0)		2 (100)		2 (100)		2 (100)	
Mitoses / 2mm^2^													
< 2	39	17 (43.6)	0.048	26 (66.7)	1	15 (38.5)	1	16 (41.0)	0.023	13 (33.3)	0.007	34 (87.2)	0.033
≥ 2	8	7 (87.5)		6 (75.0)		3 (37.5)		7 (87.5)		7 (87.5)		4 (50.0)	
Ki-67 proliferation index													
< 3%	39	18 (46.2)	0.245	27 (69.2)	0.697	15 (38.5)	1	18 (46.2)	0.461	15 (38.5)	0.258	33 (84.6)	0.167
≧3%	8	6 (75.0)		5 (62.5)		3 (37.5)		5 (62.5)		5 (62.5)		5 (62.5)	
Grade													
G1	37	16 (43.2)	0.101	26 (70.3)	0.622	15 (40.5)	0.249	16 (43.2)	0.204	13 (35.1)	0.089	32 (86.5)	0.04
G2	9	7 (77.8)		5 (55.6)		2 (22.2)		6 (66.7)		6 (66.7)		6 (66.7)	
G3	1	1 (100)		1 (100)		1 (100)		1 (100)		1 (100)		0 (0)	
Recurrence													
Negative	41	20 (48.8)	0.666	28 (68.3)	1	17 (41.5)	0.384	19 (46.3)	0.416	16 (39.0)	0.379	33 (80.5)	1
Positive	6	4 (66.7)		4 (66.7)		1 (16.7)		4 (66.7)		4 (66.7)		5 (83.3)	

### Relationships between immunohistochemical factors and prognosis

**Fig. 2:**
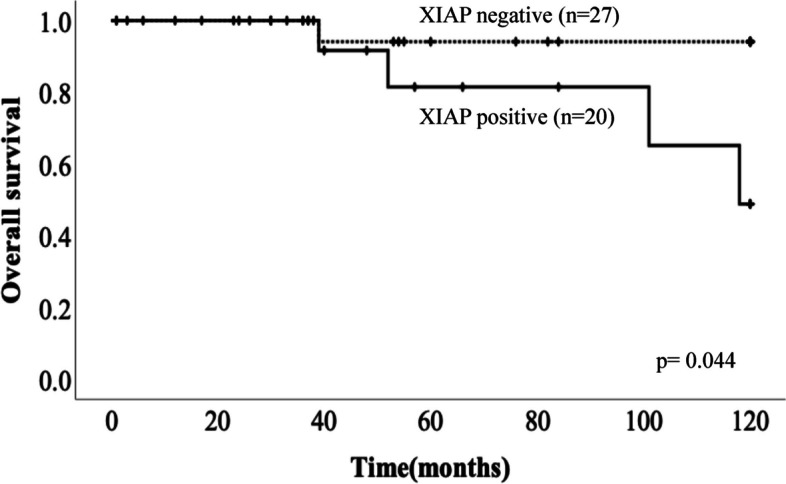
Kaplan–Meier curves for overall survival (OS) in patients with PanNET. Patients in the positive XIAP expression group showed significantly poorer OS compared with those in the negative group (*p* = 0.044)

We examined the relation between RFS and OS and the expression of GLUT1, Survivin, XIAP, Caspase3, SSTR2a, and SSTR5. Patients in the positive XIAP expression group showed significantly poorer OS compared with those in the negative group in Fig. 2 (*p* = 0.044). There were no significant differences in OS based on the positive or negative expressions of GLUT1, Survivin, Caspase3, SSTR2a, and SSTR5 in Additional file: Figure S2 (*p* = 0.276, *p* = 0.542, *p* = 0.807, *p* = 0.473, *p* = 0.845, respectively). The Kaplan–Meier curves for OS are shown in Fig. 2. On the other hand, the expression of GLUT1, Survivin, XIAP, Caspase3, SSTR2a, and SSTR5 showed no correlation with RFS (*p* = 0.548, *p* = 0.412, *p* = 0.189, *p* = 0.967, *p* = 0.767, *p* = 0.221, respectively).

## Discussion

In this study, we focused on factors related to the treatment of PanNET, with a particular focus on apoptosis. Survivin and XIAP are members of the inhibitors of IAP family and Caspase3 is an apoptosis executor. Survivin inhibits apoptosis by binding to XIAP and inhibiting the activity of Caspase3, which is one of the factors for executing apoptosis [17]. Many studies have shown that overexpression of Survivin and XIAP is found in a variety of carcinomas and is associated with poor prognosis [19, 20]. Our findings show that Survivin and XIAP expression was increased in patients with larger mitotic rate and more advanced T-stage and p-Stage. On the other hand, Caspase3 expression was opposite to that of Survivin and XIAP. There was a positive correlation between Survivin and XIAP expression. A negative correlation was found between these two proteins and Caspase3. Therefore, it is suggested that Survivin and XIAP suppress Caspase3 and inhibit apoptosis, resulting in higher malignancy in PanNET. These are the possible mechanisms suggested by this study. In other words, we can assume that the grade of malignancy correlates with the expression of Survivin and XIAP in PanNET as well as other carcinomas.

XIAP is one of the most potent endogenous inhibitors of the caspases [17] and is considered a key regulator of cell death. Apoptosis would be promoted, and anti-tumor effects could be obtained, if the function of XIAP could be inhibited. Hence, XIAP has the potential to be an ideal point for targeted therapy. A XIAP inhibitor called Embelin has been shown to have anti-tumor effects such as inhibition of cell proliferation and induction of apoptosis in vitro experiments using osteosarcoma cells [21], prostate cancer cells [22], and pancreatic cancer cells [23]. It is expected that Embelin may have anti-tumor effects in PanNET, but further detailed investigations are required to clarify the effects of Embelin on PanNET.

Yao et al. [1] and Ikeda et al. [10] discussed treatment strategies in PanNET. They both stated that therapeutic agents should be selected according to tumor burden and aggressiveness. Moreover, they mentioned that somatostatin analogue or a molecular-targeted agent should be selected for low-grade tumors, and a cytotoxic or molecular-targeted agent should be selected for high-grade tumors. Therefore, the expression of Survivin and/or XIAP may be good markers for choosing cytotoxic or molecular-targeted agents. However, we did not perform the examination using cell lines and therapeutic drugs in this study. Therefore, it is difficult to accurately determine the most optimal regimen based on the protein expression we examined in this study.

The Kaplan–Meier curves and log-rank tests showed that patients in the positive XIAP expression group had significantly lower OS than those in the negative group. Results of this study show the expression of XIAP may play a key role as a poor prognostic factor in PanNET. Several reports showed that Ki-67 and CgA evaluated using immunohistochemistry were useful prognostic markers [24]. Ki-67 is also an important indicator for the grading of PanNET. On the other hand, due to intratumoral heterogeneity, areas with a high frequency of Ki-67-positive cells and areas with a high and low frequency of Ki-67-positive cells may coexist. Evaluation of XIAP is less affected by heterogeneity because histological evaluation using XIAP confirms its expression. The expression of XIAP is considered to be an unfavorable prognostic factor in esophageal cancer [19], breast invasive ductal carcinoma [25], and salivary gland adenoid cystic carcinoma [20]. Our findings are in line with these previous studies. Therefore, the expression of XIAP may play a key role as a poor prognostic factor in PanNET. The circulating biomarkers as a prognostic factor, such as CgA, neuron-specific enolase (NSE) and pancreatic polypeptide (PP) in PanNET has also been reported. A representative circulating biomarker in PanNET is CgA [26]. The RADIANT-1 trial found that circulating CgA levels were associated with progression-free survival (PFS) and OS [27]. However, plasma CgA concentrations may fluctuate with proton pump inhibitor and renal dysfunction. Therefore, plasma CgA concentrations can sometimes be difficult to accurately assess. Despite this problem, blood tests are relatively minimally invasive and may be useful as an indicator to assess recurrence, since it is possible to monitor changes in values. The usefulness of plasma XIAP has not been studied in PanNET, and further detailed studies are expected. Liquid biopsy has been attracting attention in recent years, the NETest is a multigenomic mRNA liquid biopsy that has proved to be an accurate in vitro diagnostic for PanNET [28]. The NETest showed better performance for early diagnosis, monitoring of therapeutical efficiency, and detection of tumor relapse. It is expected that more specific markers will be added and it will become more popular in the future.

In the 5 subtypes of SSTRs, SSTR2 is the most commonly expressed SSTR in gastrointestinal NET (90%) and PanNET (80%). Somatostatin receptor scintigraphy is a modality that uses this property to diagnose neuroendocrine tumors and is now widely used worldwide. By binding to SSTR, somatostatin analogues not only suppress the secretion of endocrine hormones but also exert anti-tumor effects. In particular, its anti-tumor effects on neuroendocrine tumors have been shown by the PROMID study [29]. In our study, there was no significant difference between SSTR2a and SSTR5 expression and grade in PanNET, which suggests that SSTR2a and SSTR5 are expressed regardless of grade and that somatostatin analogues are not an agent that can be selected according to grade in PanNET. Lanreotide, a somatostatin analogue, is a synthetic peptide with affinity for SSTR2a and SSTR5. The CLARINET study revealed that lanreotide was significantly related to prolonged progression-free survival among patients with metastatic enteropancreatic neuroendocrine tumors of grade 1 or 2 [30]. All patients included in the CLARINET study were positive for SSTR expression. Therefore, it is advisable to confirm the expression of SSTR when selecting a therapeutic agent.

In the present study, the expression of GLUT1 was significantly increased in the group with high mitotic counts. The mitotic rate is one of the factors that defines tumor grade of PanNET in the WHO classification 2019. GLUT1 expression is reported to correlate with tumor aggressiveness and poor prognosis in various carcinomas such as those of the bladder, breast, and pancreas [15, 31, 32]. Moreover, Fujino et al. [33] revealed that GLUT1 expression correlated with not only mitotic rate but also tumor aggressiveness, vessel invasion, lymph node metastasis, and high Ki-67 labeling index. The only statistically significant difference in our study was in the relationship between GLUT1 expression and mitotic rate, but the results were generally similar to those of Fujino et al. These findings imply that the expression of GLUT1 can be useful in the assessment of the malignancy in PanNET. Usuda et al. [34] reported that GLUT1 expression correlates significantly with ^18^F-fluoro-2-deoxyglucose uptake on positron emission tomography in lung cancer, and this may be a useful modality for identifying lesions and evaluating distant metastases in high-grade PanNET.

This study has several limitations. The design was a single-institution retrospective analysis. The sample size was small, and the incidence of patients with G3 was less compared with those with G1 and G2 in this study. This is a notable limitation of this study. It will be necessary to accumulate as many cases as possible to eliminate grade bias. Furthermore, it is difficult to derive a correlation with progression just by seeing at the correlation between expression and worse survival. Cell lines could be used to further test and assess tumor progression, in addition to immunohistochemistry.

## Conclusions

In conclusion, the present study suggested that IAPs such as Survivin and XIAP, and GLUT1 correlated with the grade of malignancy in PanNET, and in particular, XIAP expression was associated with an unfavorable prognosis. The expression of these proteins may be a useful indicator with which to evaluate the grade of PanNET and to select optimal therapeutic agents.

### Supplementary Information

**Additional file 1: Figure S2.** Kaplan–Meier curves for overall survival (OS) in patients with PanNET. Kaplan–Meier curves for OS by expression of GLUT1, SSTR2a, SSTR5, Survivin, and Caspase3. There were no statistically significant differences in OS based on the positive or negative expressions of GLUT1, SSTR2a, SSTR5, Survivin, and Caspase3 (*p* = 0.276, *p* = 0.473, *p* = 0.845, *p* = 0.542, *p* = 0.807, respectively).

## Data Availability

The data used during the current study are available from the corresponding author on reasonable request.

## Abbreviations

CgA: Chromogranin A
DP: Distal pancreatectomy
IAP: Inhibitors of apoptosis proteins
mTOR: Mammalian target of rapamycin
NET: Neuroendocrine tumors
NSE: Neuron-specific enolase
OS: Overall survival
PanNET: Pancreatic neuroendocrine tumors
Pbt: Pancreatic body and tail
PD: Pancreatoduodenectomy
PFS: Progression-free survival
Ph: Pancreatic head
PP: Pancreatic polypeptide
PRRT: Peptide receptor radionuclide therapy
RFS: Relapse-free survival
SSTR: Somatostatin receptor
S6K1: Ribosomal protein S6 kinase 1
VEGFR: Vascular endothelial growth factor receptor
XIAP: X-chromosome-linked inhibitor of apoptosis protein
