# Supplementary figures and images for: Apoptosis-related factors are relevant to progression of pancreatic neuroendocrine tumors
Source: World J Surg Oncol. 2023 Dec 12;21:381. doi: 10.1186/s12957-023-03267-4 (PMC10714622; doi:10.1186/s12957-023-03267-4)

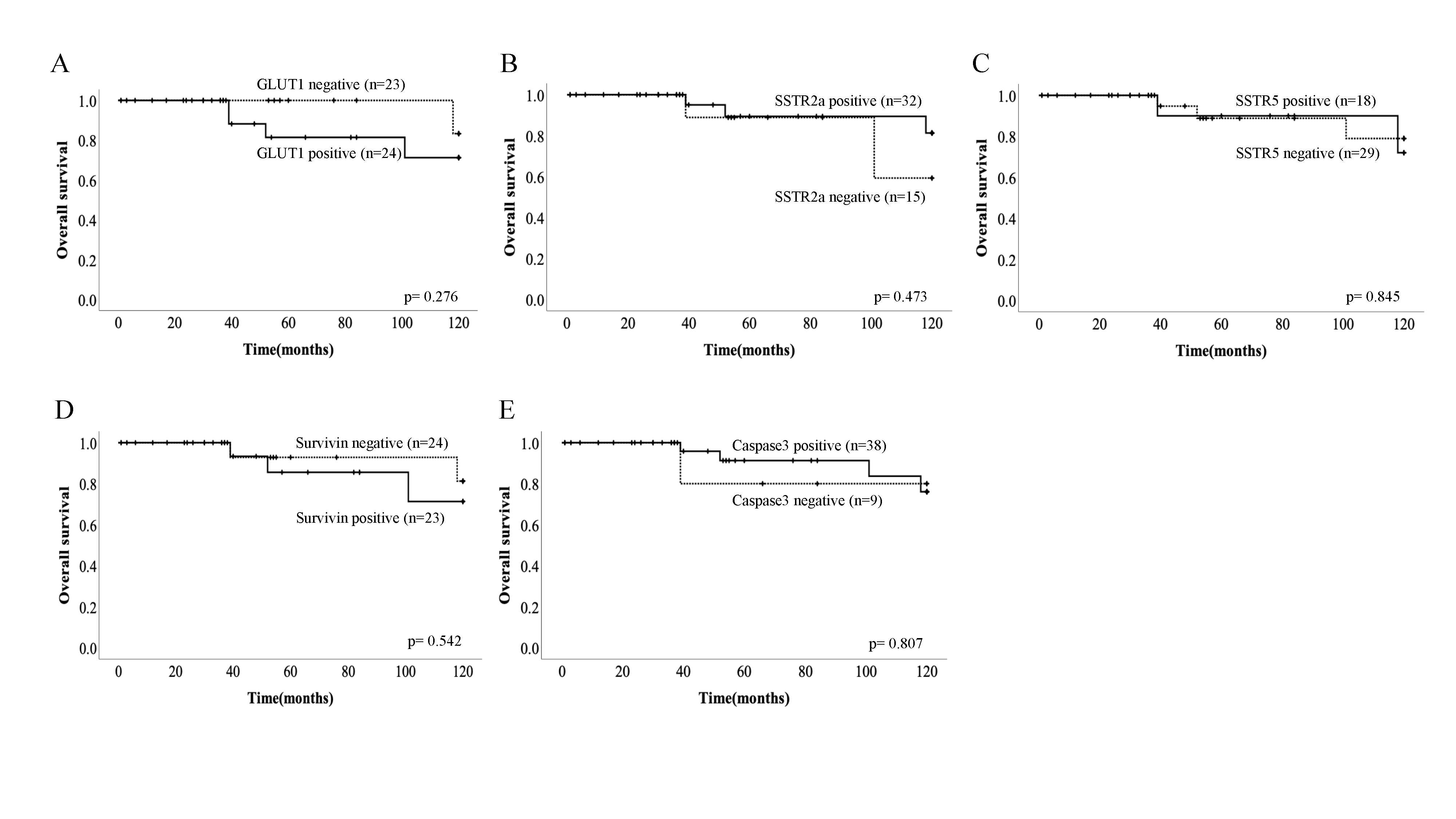

Supplement: Supplementary file 1 — Additional file 1: Figure S2. Kaplan–Meier curves for overall survival (OS) in patients with PanNET. Kaplan–Meier curves for OS by expression of GLUT1, SSTR2a, SSTR5, Survivin, and Caspase3. There were no statistically significant differences in OS based on the positive or negative expressions of GLUT1, SSTR2a, SSTR5, Survivin, and Caspase3 (p = 0.276, p = 0.473, p = 0.845, p = 0.542, p = 0.807, respectively). [file 12957_2023_3267_MOESM1_ESM.tiff]
